# Supplementary material for: Oxidised metabolites of the omega-6 fatty acid linoleic acid activate dFOXO
Source: Life Sci Alliance. 2020 Jan 28;3(2):e201900356. doi: 10.26508/lsa.201900356 (PMC6988086; doi:10.26508/lsa.201900356)
Supplement: Supplementary file 1 [file LSA-2019-00356_TableS1.doc]

Table S1. Fatty acid analysis of *Drosophila* 3^rd^ instar larvae lipid extract. Lipid extracts were analysed from *hop^Tum^* larvae grown on media supplemented with 0.15% linoleic acid. Levels of linoleic acid (C18:2n-6c), α-linolenic (C18:3n-3), arachidonic acid (C20:4n-6), eicosapentaenoic acid (C20:5n-3) and docosahexaenoic acid (C22:6n-3) are highlighted. Trace levels of odd-chain fatty acids derive from media. Data information: Data represent mean and standard deviation of three determinations.

| Fatty acid | FA % of total | FA μg/mg larvae |
| --- | --- | --- |
| C4:0 | 0.00 ± 0.00 | 0.00 ± 0.00 |
| C6:0 | 0.00 ± 0.00 | 0.00 ± 0.00 |
| C8:0 | 0.00 ± 0.00 | 0.00 ± 0.00 |
| C10:0 | 0.02 ± 0.00 | 0.04 ± 0.00 |
| C12:0 | 2.50 ± 0.10 | 5.98 ± 0.51 |
| C14:0 | 21.74 ± 1.06 | 52.20 ± 9.01 |
| C14:1 | 1.61 ± 0.16 | 3.84 ± 0.09 |
| C16:0 | 23.08 ± 0.56 | 55.34 ± 8.21 |
| C16:1 | 21.72 ± 2.63 | 51.62 ± 0.16 |
| C18:0 | 2.18 ± 0.46 | 5.28 ± 1.74 |
| C18:1n-9t | 0.00 ± 0.00 | 0.00 ± 0.00 |
| C18:1n-9c | 19.28 ± 0.17 | 46.19 ± 6.15 |
| C18:1n-7 | 0.00 ± 0.00 | 0.00 ± 0.00 |
| C18:2n-6t | 0.00 ± 0.00 | 0.00 ± 0.00 |
| C18:2n-6c | 7.00 ± 0.32 | 16.8 ± 2.86 |
| C18:3n-6 | 0.00 ± 0.00 | 0.01 ± 0.01 |
| C18:3n-3 | 0.20 ± 0.01 | 0.48 ± 0.03 |
| C20:0 | 0.25 ± 0.09 | 0.62 ± 0.29 |
| C20:1n-9 | 0.02 ± 0.01 | 0.04 ± 0.02 |
| C20:2 | 0.00 ± 0.00 | 0.00 ± 0.00 |
| C20:3n-6 | 0.00 ± 0.00 | 0.00 ± 0.00 |
| C20:4n-6 | 0.01 ± 0.00 | 0.01 ± 0.00 |
| C20:3n-3 | 0.00 ± 0.00 | 0.00 ± 0.00 |
| C22:0 | 0.06 ± 0.04 | 0.14 ± 0.01 |
| C22:1n-9 | 0.02 ± 0.01 | 0.04 ± 0.03 |
| C20:5n-3 | 0.00 ± 0.00 | 0.00 ± 0.00 |
| C23:0 | 0.00 ± 0.00 | 0.00 ± 0.00 |
| C22:2 | 0.00 ± 0.00 | 0.00 ± 0.00 |
| C24:0 | 0.00 ± 0.00 | 0.01 ± 0.01 |
| C24:1 | 0.00 ± 0.00 | 0.00 ± 0.00 |
| C22:5n-3 | 0.00 ± 0.00 | 0.00 ± 0.00 |
| C22:6n-3 | 0.00 ± 0.00 | 0.00 ± 0.00 |
